# Host Factors in the Infection Cycle of *Bamboo mosaic virus*

**DOI:** 10.3389/fmicb.2017.00437

**Published:** 2017-03-15

**Authors:** Ying-Ping Huang, I-Hsuan Chen, Ching-Hsiu Tsai

**Affiliations:** Graduate Institute of Biotechnology, National Chung Hsing UniversityTaichung, Taiwan

**Keywords:** *Bamboo mosaic virus*, host factors, virus replication, virus movement, defense proteins

## Abstract

To complete the infection cycle efficiently, the virus must hijack the host systems in order to benefit for all the steps and has to face all the defense mechanisms from the host. This review involves a discussion of how these positive and negative factors regulate the viral RNA accumulation identified for the *Bamboo mosaic virus* (BaMV), a single-stranded RNA virus. The genome of BaMV is approximately 6.4 kb in length, encoding five functional polypeptides. To reveal the host factors involved in the infection cycle of BaMV, a few different approaches were taken to screen the candidates. One of the approaches is isolating the viral replicase-associated proteins by co-immunoprecipitation with the transiently expressed tagged viral replicase in plants. Another approach is using the cDNA-amplified fragment length polymorphism technique to screen the differentially expressed genes derived from *N. benthamiana* plants after infection. The candidates are examined by knocking down the expression in plants using the *Tobacco rattle virus*-based virus-induced gene silencing technique following BaMV inoculation. The positive or negative regulators could be described as reducing or enhancing the accumulation of BaMV in plants when the expression levels of these proteins are knocked down. The possible roles of these host factors acting on the accumulation of BaMV will be discussed.

## Introduction

When a positive-sense RNA virus infects a host cell, it needs to produce its progeny and move it to the neighboring cells efficiently. In general, the entire infection cycle starts at the viral RNA entry, using the host translation system to produce the viral-specific replicase, transition the viral template from translation status to replication status, targeting the specific organelle for replication, rearrangement of the cellular membrane, recruitment of ancillary proteins to the replication site, viral RNA replication to synthesize the minus- and plus-strand RNAs, subgenomic RNA synthesis in some species, and finally, the viral-encoded movement proteins (MPs) and coat proteins accumulated for cell-to-cell movement and encapsidation, respectively. Some of the viral-encoded proteins that evolved not only fulfilled a specific role apart from amplification, but also performed counter-defense functions such as silencing suppressors against the virus-induced gene silencing system (Qu and Morris, 2005) or preventing the spread of the gene silencing signal (Voinnet et al., 2016).

*Bamboo mosaic virus* (BaMV) is a positive-sense, single-stranded RNA virus, belonging to the genus *Potexvirus* of the family *Alphaflexiviridae*. The genome of BaMV is approximately 6.4 kb in length with a 5′ m^7^GpppG structure and a 3′-end poly(A) tail and contains five open reading frames (ORFs) (Lin et al., 1994). The 3′ untranslated region (UTR) was demonstrated to form a complexed structure (including a cloverleaf-like structure, a major stem-loop, and a pseudoknot) and acted as a *cis*-acting element for minus-strand RNA synthesis, polyadenylation, an intracellular trafficking signal, and long distance movement (Cheng and Tsai, 1999; Tsai et al., 1999; Chen et al., 2005; Lin et al., 2005; Cheng et al., 2013). ORF 1 encodes replicase, including a capping enzyme domain (Li et al., 2001a; Huang et al., 2004), a helicase-like domain (Li et al., 2001b), and an RNA-dependent RNA polymerase domain (Li et al., 1998). ORFs 2-4 overlapping genes termed “triple-gene-block” (TGB) encode the MPs (TGBp1, TGBp2, and TGBp3) involved in virus movement (Lin et al., 1994). ORF 5 encodes the capsid protein (CP) for virus encapsidation, movement, and symptom development (Lin et al., 1994; Lan et al., 2010; Lee et al., 2011; Hung et al., 2014). Furthermore, a satellite RNA (satBaMV) was identified to associate with BaMV and could be amplified by BaMV replicase (Lin and Hsu, 1994; Lin et al., 2006). The tertiary structures of the 5′ and 3′ UTRs of satBaMV were revealed to be similar to those of BaMV (Huang et al., 2009; Chen et al., 2010).

As mentioned previously, the positive-sense RNA virus has to establish an efficient replication after entry into a host cell; the host factors are usually required to join and form a multi-functional replication complex (Ahlquist et al., 2003; Nagy and Pogany, 2008; Huang et al., 2012a). The replicase complex isolated from Qβ-infected cells is composed of bacterial proteins, elongation factors EF-Tu and -Ts and ribosomal protein S1, and Qβ RdRp for plus-strand RNA synthesis (Blumenthal and Carmichael, 1979; Blumenthal, 1980). Additional bacteria protein HF1, a ribosome-associated protein, is required for the complex to synthesize the minus-strand RNA (Barrera et al., 1993). The eukaryotic translational elongation factor 1a (eEF1a) was revealed to be part of the replicase complex in tobamoviruses, tymoviruses, potyviruses, and tobusviruses (Joshi et al., 1986; Mans et al., 1991; Dreher et al., 1999; Nishikiori et al., 2006; Yamaji et al., 2006; Thivierge et al., 2008; Li et al., 2010; Luan et al., 2016).

A few strategies were used to identify the host factors involved in virus infection cycles. By screening the host cDNA library constructed in yeast with the two-hybrid technique, one can discover the specific host factor that interacted with the viral-encoded target protein, such as the replicase, MPs, or CP (Ren et al., 2000; Nagy, 2008; Schoelz et al., 2011). The virus-encoded proteins can also be used as a ligand to co-immunoprecipitation the possible candidates for interaction with the host (DeBlasio et al., 2015, 2016). In the UV cross-linking competition technique, host proteins could be identified as interacting with the viral RNA, such as the 5′ or 3′ UTRs (Lin et al., 2007; Huang et al., 2012b; Hyodo et al., 2014). The identities of the interacted candidates derived from co-immunoprecipitation or UV cross-linking techniques could be revealed by LC/MS/MS. The cDNA-amplified length polymorphism (AFLP), a highly sensitive and efficient technique used for studying gene expression (Money et al., 1996) and demonstrated to deliver reproducible results (Bachem et al., 1996; Ditt et al., 2001), was used to screen the host’s differentially expressed genes in a post-virus infection (Cheng et al., 2010). The up- and downregulated cDNA fragments could be easily visualized and compared when run in parallel on the gel. These differentially expressed cDNA fragments could be straightforwardly isolated, amplified, cloned, and sequenced.

To reveal the relationship of the interacting host proteins with viral proteins or RNAs and the differentially expressed proteins in a post-virus infection, the *Tobacco rattle virus* (TRV)-based virus-induced gene silencing (VIGS) technique (Ruiz et al., 1998; Ratcliff et al., 2001) could be used to knock down the expressions and examine their effect on virus accumulation (Cheng et al., 2010). The results derived from the specific gene knockdown experiment (i.e., a loss of function) can be further confirmed by the complementary results derived from a transient expression of the same gene (i.e., a gain of function). These results would reveal whether the specific gene is playing an assistant or defense role in the virus**’** life cycle. Furthermore, the results of viral accumulation in the specific gene knockdown plants and protoplasts could specify that the host factor is acting in the replication or movement step of the infection cycle.

The following sections provide discussions of how the host factors identified by the techniques described above participate in BaMV replication and movement. The study of virus infection mechanisms and hosts’ responses to them will provide a better understanding of the relationship between pathogens and hosts. This learning would lead to designing the better strategies for pathogen control on plants.

## The Factors Involved in Assisting BaMV RNA Replication

**FIGURE 1:**
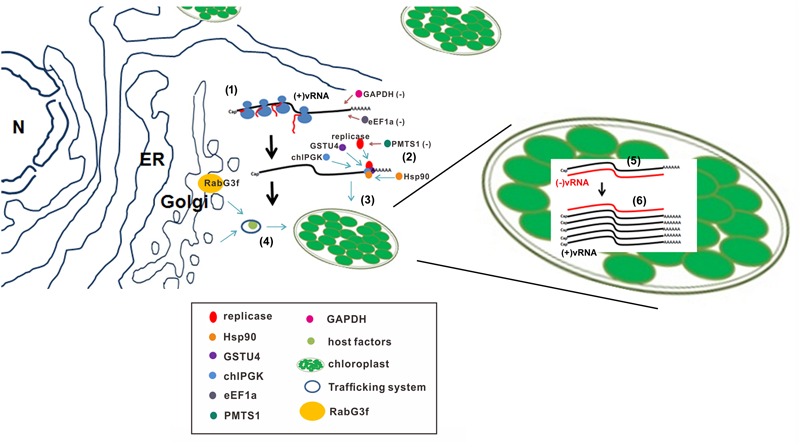
**A schematic representation of the model for BaMV RNA-replicase-host factors interaction in the replication steps.** (1) Once BaMV RNA entering the cell, the viral replicase is translated using the host translation system. (2) The 3′ untranslated region of BaMV genomic RNA shown as (+)vRNA is interacted with several host factors which regulate BaMV replication positively including chloroplast phosphoglycerate kinase (chlPGK), heat shock protein 90 (Hsp90), thioredoxin transferase GSTU4, and negatively indicated with red arrows including elongation factor 1a (eEF1a) and glyceraldehyde 3-phosphate dehydrogenase (GAPDH). Another host factor a putative methyltransferase (PMTS1) is also shown as a negative regulator for BaMV replication through the interaction with viral replicase. (3) The (+)vRNA is transported to the chloroplasts through the interaction with chlPGK for replication. (4) Some of host factors required for BaMV replication can be transported into chloroplasts by the endomembrane trafficking system using one of the Rabs, NbRabG3f. (5) The minus-strand RNA shown as (-)vRNA is synthesized inside the chloroplasts. (6) The plus-strand (+)vRNA is then synthesized following with (-)vRNA synthesis.

The entire replication processes of a positive-sense RNA virus could be divided into a few different steps. First, once BaMV enters a host cell, with bamboo as a natural host and *N. benthamiana* as an experimental host, the RNA genome is used as a template for translation to synthesize the replication enzyme, replicase (**Figure 1**). At this stage, the 3′ UTR is playing a critical role in trapping a few different factors that would lead to the next step of the replication process, targeting the replication site. At least four host proteins, glutathione transferase U4 (NbGSTU4), the eEF1a, chloroplasts PGK (chlPGK), and heat shock protein 90 (Hsp90), were discovered to interact with the 3′ UTR of BaMV. In particular, the eEF1a was shown to play a negative role in BaMV RNA replication (Lin et al., 2007). Since the binding site of the eEF1a in the 3′ UTR of BaMV is overlapped with that of the RdRp, the eEF1a might play a role in the template switch that blocks viral replication during translation. The eEF1a has commonly been demonstrated to bind the tRNA-like structure of *Brome mosaic virus* (BMV) (Bastin and Hall, 1976) and *Turnip yellow mosaic virus* (TYMV) (Joshi et al., 1986). This interaction was claimed to function in the negative-regulation of TYMV minus-strand RNA synthesis (Matsuda et al., 2004); however, a similar interaction in *West Nile virus* was revealed to facilitate minus-strand RNA synthesis (Davis et al., 2007).

The nucleus-encoded chlPGK interacting with the 3′ UTR (Lin et al., 2007) was demonstrated to play a role in ushering the viral RNA and its associated proteins, including replicase, into chloroplasts for replication (**Figure 1**) (Cheng et al., 2013). The genomic RNA was visualized in the chloroplast by confocal microscopy after being labeled with a green fluorescent protein fusion MS2 coat protein construct (NLS-MS2-GFP) that could recognize the BaMV RNA containing the MS2 hairpins (Cheng et al., 2013). The advantage of BaMV targeting a chloroplast for replication is that it might be a way to hide from the host scavenging system, including the RNA silencing pathway. To get into the chloroplast for replication, the entire replication complex—including the viral RNA [approximately 6.4 kb plus the poly(A) tail], replicase, and other associated host factors—must be transporting into the chloroplasts through the chloroplast transporting complex. This gigantic viral RNA-protein complex can enter into the chloroplasts because Hsp90 interacting with the 3′ UTR of BaMV was demonstrated to play a positive role in the very early event of BaMV replication (Huang et al., 2012b). Heat shock proteins acting as chaperones on protein complex folding, protein degradation, and protein translocation across membranes (Mayer and Bukau, 2005; Taipale et al., 2010) could help transport the viral RNP complex into the chloroplasts (**Figure 1**). HSPs were shown to be assisting the viral RNA recruitment and viral replication complexes (VRCs) assembly (Pogany et al., 2008; Wang et al., 2009a,b; Huang et al., 2012b). Accordingly, Hsp90 involved in the early event of BaMV replication could be implied to assist the viral RNP complex entry into the chloroplasts and stimulate the replication complex assembly on the right location in order to initiate the minus-strand RNA synthesis.

Another 3′ UTR-associated protein, NbGSTU4, was demonstrated to be upregulated post BaMV infection and involved in assisting the replication of BaMV *in vitro* and *in vivo* (Chen et al., 2013). In general, GSTs are involved in the antioxidation process; the oxidative stress triggered by a pathogen infection could be attenuated via the enzymatic reaction of GSTs. A chloroplast is one of the major reactive oxygen species (ROS) producer in which the relative concentration of ROS should be higher than that of other organelles. Once BaMV enters into the chloroplasts for replication, the viral RNAs face the obstacle of the higher levels of ROS produced either from the photosynthesis process or the virus infection. NbGSTU4 moving with BaMV RNA in the presence of Glutathione (GSH) could play a role in eliminating the effects of ROS. We have demonstrated that NbGSTU4 could interact with the 3′ UTR in the physiological concentration of GSH, which is approximately 10 mM (Chen et al., 2013). These results imply that NbGSTU4 could also be one of the host proteins associated with viral RNA and was transported together into the chloroplasts.

A 5′ to 3′ exonuclease (XRN4), a protein component enriched in BaMV RdRp preparation, was demonstrated to enhance the accumulation of BaMV (Lee et al., 2015). The members of the XRN_N family are divided into two groups, the cytoplasmic (XRN1/PACMAN or XRN4) and the nuclear enzymes (XRN2/RAT1 and XRN3), and display the RNase activities for mRNA degradation (Kastenmayer and Green, 2000; Souret et al., 2004). Although XRN4 was revealed to conduct antiviral activity against *Tomato bushy stunt virus* (TBSV) and *Tobacco mosaic virus* (TMV) (Cheng et al., 2007; Jaag and Nagy, 2009; Peng et al., 2011), the presence of XRN4 could elevate the accumulation of BaMV. Because XRN4 was demonstrated to have a role in reducing the activities of siRNA- and miRNA-mediated RNA decay in *Arabidopsis* (Souret et al., 2004), downregulated the silencing pathway might result in a positive regulation of BaMV RNA accumulation.

Some host factors identified by cDNA-AFLP could influence the replication of BaMV in an indirect manner so that these proteins could not be revealed by using the strategy of detecting the direct interaction with viral products. NbRabG3f was demonstrated to be a positive regulator in BaMV replication by loss- and gain-of-function assays (Huang et al., 2016). Rabs are a group of small GTPases involved in vesicles transport, uncoating, tethering, and fusion (Seabra et al., 2002). A deletion mutant of NbRabG3f failure in membrane-anchoring lost the ability to assist the accumulation of BaMV. A mutant with the fixed GDP-bound RabG3f (T22N) was trapped at the Golgi and could not assist the accumulation of BaMV. Overall, these results suggest that NbRabG3f is involved in a vesicle budding from the Golgi and transports the cargos containing the unidentified host factors to the destination site for BaMV replication (**Figure 1**).

Based on the host factors identified so far for BaMV replication, BaMV RNA entry into the host cell would require chlPGK to usher the viral RNA into the chloroplasts. The transport of the viral RNP complex needs the chaperon Hsp90 to cross the membrane and assemble the functional replication complex. During this process of trafficking from the cytoplasm to the chloroplasts, NbXRN4 might be involved in reducing the activities of siRNA-mediated silencing. Once the viral RNP is transported into the chloroplasts, the VRC needs the anti-oxidant enzyme NbGSTU4 to neutralize the oxidative stress inside the chloroplasts for an efficient minus-strand RNA synthesis (**Figure 1**).

## The Factors Involved in Viral RNA Movement

Through a biochemical analysis of the BaMV movement complex isolated by co-immunoprecipitation using an anti-TGBp3 antibody, the movement trafficking complex was revealed to harbor not only the MPs TGBp1, TGBp2, and TGBp3, but also the coat protein and replicase (Chou et al., 2013). The TGBps-mediated cell-to-cell trafficking was proposed to be in two possible paths: TGBps-associated virion complex traffics alongside the endoplasmic reticulum (ER) network, or the virions and the MPs would associate with the TGBp2-induced vesicles (Chou et al., 2013; Liou et al., 2015).

**FIGURE 2:**
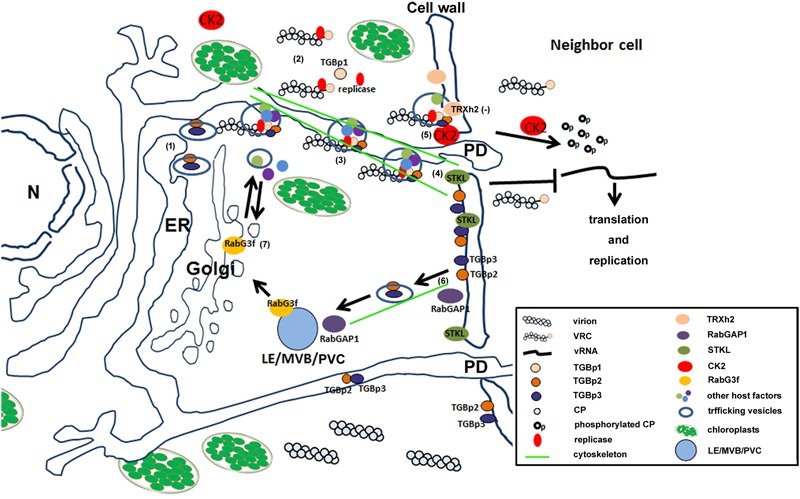
**A schematic view of the hypothetical model for BaMV movement.** The intracellular trafficking of BaMV movement complex is proposed to form the vesicle and trafficking through the cytoskeleton toward the plasmodesmata (PD). The steps of the BaMV movement are illustrated as (1) the movement proteins of BaMV, TGBp2 and TGBp3, are synthesized on the endoplasmic reticulum (ER) and transported via vesicles possibly regulated by one of the Rab-GTPases; (2) the newly synthesized viral RNA is assembled with the capsid protein (CP), movement protein TGBp1, and the viral replicase to form a competent viral replication complex (VRC); (3) the VRC and possibly some other host factors are recruited to TGBp2/TGBp3-containing vesicle to form a movement complex and trafficking toward the PD; (4) the host factor STKL localized on the plasma membrane might control the gate of the PD; (5) the host factor CK2α targeting the CP of VRC and release the vRNA from the VRC to the neighbor cell for further translation or replication; (6) TGBp2 and TGBp3 are released from the movement complex after disassembly on the PD and might shuttle back from the plasma membrane to late endosome/multivesicle bodies/prevacuolar compartments (LE/MVB/PVC) with the help of RabGAP1 to activate the Rab (unidentified yet); (7) RabG3f is possibly involved in shuttling the viral movement proteins back to Golgi and ER. One of the host factors, TRXh2, is shown to play a negative role indicated as (-) in hindering the movement through the interaction with viral movement protein TGBp2.

A few host factors were identified to participate in the process of BaMV movement. A RabGTPase-activating protein (GAP) designated as NbRabGAP1 was demonstrated to participate in BaMV cell-to-cell and systemic movements (Huang et al., 2013). Rabs, a family of small GTPases, are known to be involved in all aspects of intracellular vesicle budding, targeting, docking, and fusion (Johansen et al., 2009; Mizuno-Yamasaki et al., 2012; Cherfils and Zeghouf, 2013). Two Rabs regulators, guanine nucleotide exchange factors (GFFs), and GAPs play roles in recycling Rabs for vesicles trafficking, in which GEFs exchange GDP for GTP and GAPs accelerate GTP hydrolysis (Bos et al., 2007). The results of the mutational analysis of NbRabGAP1in BaMV accumulation suggest that the fully GAP function of NbRabGAP1 is essential to support the efficient movement of BaMV. The proposed role of NbRabGAP1 in BaMV movement is that NbRabGAP1is to trigger one of the RabGTPases (not yet identified) to release the vesicles containing the viral movement complex trafficking to the plasmodesmata (PD) (**Figure 2**), similar to those revealed in *Chinese wheat mosaic virus* (Andika et al., 2013), or to shuttle TGB proteins from the PD via the endocytotic pathway back to the ER, like those found in *Potato mop top virus* (Haupt et al., 2005).

A serine/threonine kinase-like protein from *N. benthamiana* (NbSTKL), an upregulated gene that is post BaMV inoculation, was demonstrated to be critical in the movement step of the BaMV infection cycle (Cheng et al., 2013). The results from the sequence analysis and the intracellular localization indicated that NbSTKL is plasma membrane-associated through myristoylation at glycine, the second amino acid from the N-terminus. The mutant that lost the kinase activity (NbSTKL/D224A) or failed to associate with the plasma membrane (NbSTKL/G2A) also failed to enhance the movement of BaMV. These results suggest that NbSTKL might target a specific factor on the membrane, regulating the gating of the PD for the passage of BaMV (**Figure 2**). Furthermore, another kinase, casein kinase 2α (CK2α), which interacts with BaMV CP in PD, might assist the release of viral RNA from the RNP movement complex during the virial RNP complex passage through the PD (**Figure 2**) (Hung et al., 2014).

Taken together, the identification of NbRabGAP1 involved in the movement of BaMV supports the idea that the movement could be made through the vesicle trafficking path (Chou et al., 2013; Liou et al., 2015). To reach an efficient movement of BaMV requires at least two kinases, NbSTKL and CK2α, gating the PD and releasing the viral RNA from the RNP complex (**Figure 2**). Obviously, some other factors are also required for this process such as the target of NbSTKL.

## The Factors Involved in Defense Against Viral RNA Replication

As mentioned previously, once a virus enters a host cell, it needs not only to seek the host factors for assistance, but also to face the challenges from the host itself. In plants, there already exist a few defense mechanisms such as the RNA silencing pathway and the elector-induced hypersensitive reaction. In addition, some novel proteins in host cells could display anti-viral activities beyond their already known functions. A putative methyltransferase (PMTS1) once interacted with BaMV RdRp, screened by a yeast two-hybrid technique, and displayed an inhibitory effect on the RdRp activity with a dosage-dependent fashion (**Figure 1**) (Cheng et al., 2009). PMTS1 comprises an N-terminal signal peptide predicted to target mitochondria or chloroplasts and two putative AdoMet-binding motifs in the middle region. Removing the signal peptide or abolishing the AdoMet-binding activity of PMTS1 would cause the loss of its inhibitory effect. Because BaMV has been demonstrated to replicate in chloroplasts, the signal peptide of PMTS1 targeting the chloroplasts is highly recommended.

Glyceraldehyde 3-phosphate dehydrogenase (GAPDH) found in the purified RdRp complexes could bind the stem-loop C/poly(A) in the 3′ UTRs of satBaMV and the pseudoknot/poly(A) in the 3′ UTR of BaMV (Prasanth et al., 2011). Further analysis indicates that the cytosolic, GAPDH, could inhibit the replication of BaMV/satBaMV (**Figure 1**). The purified recombinant, GAPDH, could specifically inhibit the synthesis of minus-strand RNA of BaMV/satBaMV in an *in vitro* replication assay. GAPDH is a multifunctional enzyme involved in quite diverse activities in cells, including glycolysis, cellular dysfunction, cell death, apoptosis, association with cytoskeleton and vesicles transport, exportation of nuclear RNA, and DNA repair (Tristan et al., 2011). In *Arabidopsis*, cytosolic GAPDH was found to be a prominent target of H_2_O_2_-dependent oxidation (Hancock et al., 2005), but could be reversible back in the presence of reductant GSH (Bedhomme et al., 2012). Although the functions of GAPDH involved in BaMV replication are not clear, the simple interaction of GAPDH with the 3′ UTRs of BaMV and satBaMV could block the accessibility of RdRp for initiating the minus-strand RNA synthesis.

## The Factors Involved in Defense Against Viral RNA Movement

Regarding the movement of potexviruses, both MP and CP are vital for efficient cell-to-cell movement and vascular transport (Verchot-Lubicz, 2005; Verchot-Lubicz et al., 2010). Post-translational modification, including ubiquitination, sumoylation, glycosylation, and phosphorylation of viral proteins, were issued as parts of an important process in modulating the structures and functions of viral proteins (Barajas and Nagy, 2010; Alcaide-Loridan and Jupin, 2012; Mathur et al., 2012; Perez Jde et al., 2013; Samuilova et al., 2013; Xiong and Wang, 2013). Maintaining the protein structural integrity, including those modifications of MP and CP, is critical for virus movement.

One of the thioredoxin proteins, NbTRXh2, an upregulated gene post BaMV inoculation, was demonstrated to restrict the movement of BaMV (Chen et al., 2017). NbTRXh2 was localized at the plasma membrane through myristoylation at the Glycine of the second amino acid from the N-terminus. NbTRXh2 was revealed to target the MP TGBp2 to reduce its disulfide bond (**Figure 2**). Also, the two conserved cysteins forming the disulfide bond were demonstrated to play a key role in BaMV movement (Tseng et al., 2009). Therefore, NbTRXh2 targets TGBp2, which could result in the loss of the structural integrity of TGBp2 and their failure to interact with other movement-associated proteins, including TGBps1 and 3.

## Summary and Future Prospective

Taking all the available results into account, it can be concluded that some of the host factors are unique to BaMV, while some of them could be applied to other viruses. Some host factors could assist virus replication and movement, but some of them are involved in resisting virus infection. This review summarized a few host factors identified with different strategies and their possible roles in BaMV infection. However, to complete an accurate understanding of BaMV infection, more host factors need to be identified. Based on our current understandings, a few processes in BaMV infection cycle are still unclear. One of the most challenges is to uncover the process of how the newly synthesized RNAs are transported out of the chloroplasts where BaMV replicates. Hopefully, a much clearer picture of the infection cycle of BaMV can be obtained in the near future by knowing how of these factors involved.

## Author Contributions

Y-PH wrote the viral replication part of the manuscript. I-HC wrote the viral movement part of the manuscript. C-HT wrote the rest part and edits the manuscript.

## Conflict of Interest Statement

The authors declare that the research was conducted in the absence of any commercial or financial relationships that could be construed as a potential conflict of interest.
